# Kv2.1 negatively regulates Reissner fiber development

**DOI:** 10.3389/fcell.2025.1720752

**Published:** 2026-01-05

**Authors:** R. Rosa Amini, Ruchi P. Jain, Justyna Jędrychowska, Vladimir Korzh

**Affiliations:** International Institute of Molecular and Cell Biology, Warsaw, Poland

**Keywords:** ampulla terminalis, CD133/prom1a, flexural organ, KV2.1, midline floor plate, roof plate, Scospondin, subcommissural organ

## Abstract

**Introduction:**

The potassium voltage-gated channel Kv2.1 plays a crucial role in the development of the brain’s ventricular system. Defects in the development of this system affect the formation of the Reissner fiber, a rope-like structure produced by the flexural and subcommissural organs that secrete Scospondin.

**Methods:**

The development of the Reissner fiber has been studied during normal development and in zebrafish mutants deficient in activity of the two Kv2.1 subunits – Kcnb1 and Kcng4b using a combination of immunohistochemistry and transgenic lines expressing EGFP in the subcommissural organ and floor plate.

**Results:**

The Reissner fiber develops in stages. First, the midline floor plate cells, originating from the embryonic organizer, secrete Scospondin, forming the posterior Reissner fiber. This allows us to define the posterior Reissner fiber as the acellular derivative of the embryonic organizer. The fiber separates from the floor plate, beginning in the hindbrain and extends through the neural tube, from the most anterior floor plate (i.e. the flexural organ) anteriorly to the ampulla terminalis. Second, the subcommissural organ, which is derived from the anterior roof plate, begins secreting Scospondin. This forms the anterior Reissner fiber, which spans the cerebral aqueduct. Third, the anterior Reissner fiber connects to the flexural organ, where the two fibers fuse. Fourth, after the floor plate ceases to express Scospondin, the Reissner fiber derived from the subcommissural organ replaces the transient posterior fiber derived from the floor plate. Like the subcommissural organ, the flexural organ is an attachment point for the Reissner fiber. Reissner fiber assembly involves the formation of individual microfilaments that fuse in several steps to form the single fiber.

**Discussion:**

Analysis of zebrafish mutants of Kv2.1 subunits (Kcnb1 and Kcng4b) revealed that Kv2.1 negatively regulates Scospondin production at several levels. These mutations have opposing effects on the transcript levels of several genes involved in Reissner fiber development (*sspo*, *lgals2*, and *chl1a/camel*), affect the subcommissural organ and microfilament formation, and impact Reissner fiber assembly.

## Introduction

The basic structure of the brain ventricular system (BVS) has remained largely unchanged throughout vertebrate evolution although the number of brain ventricles varies (Lowery and Sive, 2009). It increases in parallel with increase of brain complexity, from hemichordates to mammals (Fame et al., 2020). The BVS consists of a series of interconnected cavities filled with cerebrospinal fluid (CSF) and surrounded by neuroepithelial ependymal cells. It plays a crucial role in neurogenesis and brain homeostasis by regulating the flow of CSF. The neuroepithelial lining of the BVS consists of several cell types, including tanycytes, ependymocytes, CSF-contacting neurons, macrophage-like supraependymal cells, and radial glia (Del Bigio, 2024). These cells express ion and water channels, as well as cell adhesion extracellular matrix proteins and components. During embryogenesis, the roof and floor plates are two opposing signaling centers along the dorsal and ventral midlines of the BVS. The anterior region of the roof plate develops into the subcommissural organ (SCO), and the anterior floor plate forms the flexural organ (FO) (García-Lecea et al., 2017; Kiecker, 2018). The midline floor plate originates from the embryonic shield, also known as the zebrafish organizer (Shih and Fraser, 1996). Both the SCO and FO express Scospondin (Sspo), a giant matricellular protein that comprises the Reissner fiber. The Reissner fiber is an acellular filamentous structure found along the neural tube from the third ventricle to the end of the spinal cord. Several other genes (*clu, lgals1, chl1a/camel, cyc,* and *oep*), which are expressed by the ependyma, roof plate, and floor plate, as well as low density lipoproteins from CSF have been associated with RF formation (Cantaut-Belarif et al., 2018; Driever, 2018; Lehmann and Naumann, 2005; Meiniel et al., 2008; Muñoz et al., 2019; Sepúlveda et al., 2021; Troutwine et al., 2020; Vera et al., 2015; Yang et al., 2021). Despite this progress, the complete molecular composition of the RF and the RF self-assembly mechanism remain unresolved (Muñoz et al., 2019).

In zebrafish, BVS is formed by the cavitation of the neural tube due to the input of ion channels causing water influx (Lowery and Sive, 2009; 2005). Dysregulation of these channels can result in neurodegenerative diseases, including microcephalia and hydrocephalus (Sakka et al., 2011; Tully and Dobyns, 2014; Zhang et al., 2006). Animal models of BVS defects have been employed to investigate disturbances in CSF circulation and alterations in neuroepithelial integrity (Chae et al., 2004; Chang et al., 2012; Jiménez et al., 2001).

The voltage-gated potassium channel Kv2.1 is a tetramer consisting of two subunits with opposing functions. Kcnb1 is electrically active, while Kcng4 is modulatory (Bocksteins and Snyders, 2012; Vacher et al., 2008). An appropriate balance of these activities is necessary for channel function, including intracellular protein transport; the formation of endoplasmic reticulum (ER) - plasma membrane junctions; the maintenance of the plasma membrane potential; the formation of cholesterol-enriched lipid rafts; and interaction with integrins (Bortolami et al., 2023; Deutsch et al., 2012; Forzisi and Sesti, 2022; Johnson et al., 2019; Tamkun et al., 2007). Due to its connection to the intracellular skeleton, Kv2.1 activity is mechanosensitive to the physiological perturbation of the cell membrane. This is consistent with the idea that membrane tension regulates ion flow through the channel (Schmidt et al., 2012).

Kv2.1 heterozygous mutations have been associated with epileptic encephalopathy in humans (Bar et al., 2020; Jędrychowska and Korzh, 2019; Srivastava et al., 2014; Torkamani et al., 2014). Developmental defects affecting Kv2.1 subunits in homozygous mutants have been studied in mice and zebrafish. Studies in mammals have suggested roles for Kcnb1 in insulin secretion and behavior and for Kcng4 in spermatogenesis (Jacobson et al., 2007; Regnier et al., 2017; Speca et al., 2014). Zebrafish studies have shown an antagonistic role for Kv2.1 subunits in the development of hollow structures, such as the brain and ear (Jedrychowska et al., 2021; Jędrychowska et al., 2024; Shen et al., 2016).

The *kcnb1* ^*sq301*^ mutant phenotype in zebrafish is incompletely penetrant. Some mutant embryos exhibit delayed epiboly and gastrulation failure, while others undergo gastrulation and display the late phenotype of reduced hollow organs, including the brain ventricles and ears (Jedrychowska et al., 2021; Jędrychowska et al., 2024; Shen et al., 2016). In contrast, the *kcng4b* ^*sq300Gt*^ zebrafish mutant develops hydrocephalus, which closely resembles the phenotype of *hyh* mice lacking α-SNAP, a component of intracellular protein trafficking mechanism (Pérez-Fígares et al., 1998; Shen et al., 2016; Wagner et al., 2003). Kcnb1 transports Kcng4 from endoplasmic reticulum to plasma membrane, i.e., similar to α-SNAP Kcnb1 is involved in intracellular protein trafficking (Bocksteins et al., 2014). Previous studies have shown that BVS defects in *hyh* mice are associated with abnormal RF development (Pérez-Fígares et al., 1998; Wagner et al., 2003). Therefore, we expanded our analysis of BVS in Kcnb1-Kcng4b mutants to focus on the roles of SCO and FO in RF development.

## Materials and methods

### Animals

Zebrafish (*Danio rerio*) were maintained according to established protocols (Westerfield, 2007) in the Zebrafish Core Facility at the International Institute of Molecular and Cell Biology in Warsaw. This facility is licensed for breeding and research (PL14656251, registry of the District Veterinary Inspectorate in Warsaw; 064 and 051: registry of the Ministry of Science and Higher Education). The experiments involving zebrafish embryos, larvae, and adults were conducted in accordance with the European Communities Council Directive (63/2010/EEC). The developmental stages in hours post-fertilization (hpf) are based on (Kimmel et al., 1995). The *kcnb1* ^*sq301*^ (also referred to as *kcnb1* ^*−/−*^) mutant has been previously described (Shen et al., 2016). *kcng4b* ^waw304^ and *kcng4b* ^waw305^ mutants have been described previously (Gasanov et al., 2021; Jędrychowska et al., 2024). The reduced fertility of *kcng4b* ^waw305^ mutants has prevented the analysis of gene expression level.

The transgenic lines used in this study served as *in vivo* markers: the ET33-mi2A transgenic line (sqet33mi2AEt) carries the transposon Tol2 insertion in *CD133*/*prom1a* positionIt expresses cytosolic GFP in the RP, SCO, ear, *etc*. The ET33-E20 (sqet33e20Et) transgenic line carries the transposon insertion in in between *zic3* and *zic6* genes and expresses cytosolic GFP in the choroid plexus, etc. The ET33-10 (sqet3310Et) transgenic line carries the transposon insertion in the *grip2b* intron (García-Lecea et al., 2017; Parinov et al., 2004). The *scospondin-GFP* ^*ut24*^ transgenic line is a knock-in allele, where the C-terminal portion of the *scospondin* gene was precisely tagged with the GFP coding sequence (Troutwine et al., 2020).

### Microscopy

Live imaging: Zebrafish embryos were raised in E3 medium (2.5 mM NaCl, 0.1 mM KCl, 0.16 mM CaCl_2_, and 0.43 mM MgCl_2_) with the addition of 0.2 mM 1-phenyl-2-thiourea (PTU, Merck, Germany) to block pigmentation. At selected developmental stages, the embryos were manually dechorionated and anesthetized with 0.02% tricaine (Sigma-Aldrich, United States). Then, they were oriented after embedding in 2% methylcellulose (Merck, Germany) on the glass slides. A research stereomicroscope SMZ25 (Nikon, Japan) was used for imaging. The light-sheet fluorescent microscopy was performed as previously described (Jedrychowska et al., 2021). Zeiss Lightsheet Z. 1 microscope with W Plan-Apochromat objective (20x/1.0 UV-VIS) was used.

For fixed specimens, 0.8% low-melting agarose in PBS was used instead of E3 medium with 0.02% tricaine. Zeiss Lightsheet Z. 1 microscope with W Plan-Apochromat objective (40x/1.0 UV-VIS or 63x/1.0 UV-VIS) was used. Transmitted LED light was used to obtain high-resolution bright-field images. The data were saved in the LSM or CZI format and processed using ZEN (Zeiss) or ImageJ 1.51n (Fiji) software. Maximum intensity or sum slice projections were generated for each z-stack. Brightness and contrast adjustments, as well as resizing were performed using FastStone viewer 7.4 (FastStone Soft). SCO area in Figure 3 was normalized to the head–MHB distance to correct for embryo size variation.

### Immunohistochemistry

Embryos were fixed in 4% paraformaldehyde prior to staining. Embryos were stained using two-color immunohistochemistry for RF with a polyclonal rabbit AFRU antibody (1:1000), a gift of Drs. J. Grondona [Malaga, Spain], E. Rodriguez, and M. Guerra [Valdivia, Chile]) according to the described protocol (Korzh et al., 1998). The detected GFP was expressed in transgenic embryos.

### Image quantification

Embryos were scored for the shape of the SCO apical surface in the unified analysis cohort (n = 15 embryos per genotype, except *kcng4b* ^*waw305*^) used for Figure 5; Table 1. SCO openings were classified as concave, flat, convex. Frequencies were recorded as counts and percentages. Statistical analysis was performed using a Chi-square test of independence across genotypes, followed by pairwise Fisher’s exact tests.

**TABLE 1:** qRT-PCR Primers.

N	Genes	qRT-PCR primers, 5'- > 3′
1	*chl1*	TGCAGCATTCGTGCTCAACGT AACAGCCGATGAGGACAAGCA
2	*clusterin*	TCGCAAGTTGGTGAGAAATAC GGAGGCTGTGAAACAAGGAGT
3	*foxa*	AGACAGAATCAGCAGAGATGG CGGAGTCAGGGTGTAAAGC
4	*foxa3*	ATGTCCAACGAGCAGAAGATG GTAGTAGGCACCAGAGTCAATG
5	*gli1*	AGCAGCAGCAGCATTCAG AGGTGTTCTAAGGCATTGGAC
6	*lgals2a*	GGAGCACAGAGACAACAAC ATCCTGACTTCGCCTTCG
7	*lgals2b*	CAACAACTCAGCAAGATGG TGAAGTGAAGAGCGATGG
8	*shha*	GAGAGTGCTGCTGGTGTC CTGCTTGTAGGCGAGAGG
9	*spon1a*	GTGACCAAGAGGAGGATTATGCT TGTCATGAATTGCGTGTCTTCCT
10	*sspo*	CAGTGTGGATTGTGATGTG CAGAGCAGAAGCGATACC

Anterior RF length was measured in Fiji using the segmented line tool, from the SCO apical margin to the anterior tip of FO. Values were expressed as mean ± SD. n = 6 embryos per genotype were analyzed, selected from the imaging cohort based on QC criteria requiring clear lateral views with the entire SCO–FO span visible. Group differences were tested by one-way ANOVA.

SCO area was normalized to the head–MHB distance to correct for embryo size variation. Fusion node density was calculated as the number of junctions per 50 µm filament length between the SCO and FO. The SCO–fusion distance was measured as the linear span from the SCO apical surface to the nearest fusion node. Six embryos per genotype were analyzed, chosen for high-quality dorsal views that permitted accurate quantification. Values were reported as mean ± SD. Correlations between node density and SCO–fusion distance were evaluated using Pearson’s correlation coefficient.

AFRU + signal in the ampulla terminalis was extracted using ImageJ following thresholding of representative lateral projections. The AFRU-positive area (µm^2^) was measured for each embryo and expressed as mean ± SD. n = 6 embryos per genotype were included, selected for consistent lateral orientation and sufficient staining quality. Statistical analysis was performed using one-way ANOVA followed by Tukey’s *post hoc* test. Quantification reflects the total AFRU-positive area (µm^2^) within the ampulla terminalis, not integrated fluorescence intensity. Measurements capture spatial distribution rather than overall brightness of the AFRU signal.

### Cryosectioning

The embryos were cryo-sectioned, and stained immunohistochemically as described previously (Korzh et al., 1993)using anti-rabbit AFRU antibody (1:1000; gift of Drs. J. Grondona (Malaga, Spain), E. Rodriguez, and M. Guerra (Valdivia, Chile), secondary Alexa Fluor 488 tagged donkey anti-rabbit antibody (1:1000; Invitrogen, United States) and Alexa Fluor 594 phalloidin (1:200; Life Technologies, United States) for F-actin staining. Confocal imaging was performed using the LSM 800 microscope (Zeiss, Germany).

### qRT-PCR

qRT-PCR was performed according to the manufacturer’s instructions using the SsoAdvanced Universal SYBR Green Supermix and CFX Connect real-time PCR system (Bio-Rad, United States). Total RNA was extracted from 20 to 50 zebrafish embryos (except *kcng4b* ^waw305^ homozygotes due to low fertility) at 28, 48, and 72 hpf using the TRIzol-chloroform RNA extraction protocol (Sigma-Aldrich, United States). RNA was quantified using a NanoDrop™ 2000 spectrophotometer (Thermo Scientific, United States), and cDNA was synthesized from 1 μg of the total RNA using the protocol provided with the iScript™ Reverse Transcription kit (Bio-Rad, United States). Gene-specific primers for *eef1a1l1* (a housekeeping gene) and primers specific for other genes (www.zfin.org) (Table 1) were used to amplify the mRNA of interest, which were selected based on the results of the RNA-seq analysis detailed above.

Bio-Rad CFX Maestro software automatically determined the threshold cycle (Ct) of each target and reference gene amplification in control and mutant embryos. Normalized expression values were obtained directly from the software using the ΔΔCt method, with wild-type samples serving as the reference condition. Expression values were normalized to wild-type controls at each developmental stage to account for shifts in tissue composition between 24 hpf and 72 hpf, when *sspo* expression transitions from the floor plate to the SCO. This stage-specific normalization ensures that mutant versus control comparisons remain biologically meaningful. To clearly represent the data, all qPCR graphs were provided with a dotted line at the 1.0 ratio, which corresponds to wild-type expression. The data were analyzed at the normalized expression values without further transformations (e.g., 2^(-ΔΔCt)^). Statistical significance was determined using a one-way ANOVA with Tukey’s *post hoc* test for multiple comparisons; each time point was compared to the wild-type control. Comparisons between experimental groups and wild-type controls at the relevant time point are indicated by statistical significance markers (***, **, *, n.s.). Error bars show the standard error of the mean (SEM) from biological replicates. To ensure that the efficiency values remained within the ideal range of 90%–110%, the reaction efficiency (E-value) was determined independently for each gene examined. Melting curve analysis and agarose gel electrophoresis were used to verify product specificity.

## Results

### The formation of Reissner fiber is a multistep process

**FIGURE 1:**
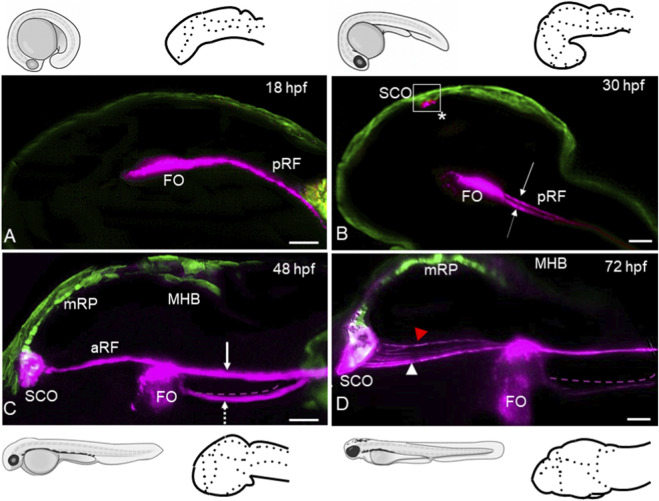
RF develops in three major steps. First, posterior RF is formed by the midline floor plate. Second, anterior RF is formed by SCO. Third, posterior RF and anterior RF fuse at FO. **(A)** In the 18 hpf embryo, anterior FP developed anti-AFRU antigenicity (magenta). **(B)** At 30 hpf, SCO developed the anti-AFRU antigenicity (asterisk). The duplicated signal was present at the level of the anterior hindbrain. **(C)** At 36 hpf, the FO acquired its characteristic shape with most of the staining dorsally. The separation of the two signals continued posterior to the FO. Additional staining corresponding to the anterior RF was detected between the SCO and FO. The signal was more intense anterior to the FO, while the region adjacent to the SCO was weakly positive for AFRU staining. **(D)** At 72 hpf, the intensity of anterior RF staining increased; several SCO-derived branches of the anterior RF formed; and the SCO and FO became elongated. For 48 hpf wild-type control, see A, A’. All images are in lateral view. The cartoons illustrate the stage of development and schematics of the brain. Solid arrows indicate trajectories or branches of the aRF; dashed arrows indicate duplicated AFRU^+^ signals posterior to the FO. Abbreviations: aRF, anterior Reissner fiber; FO, flexural organ; MHB, mid-hindbrain boundary; mRP, midbrain roof plate; pRF, posterior Reissner fiber. N = 6. Scale bar - 20 μm.

**FIGURE 2:**
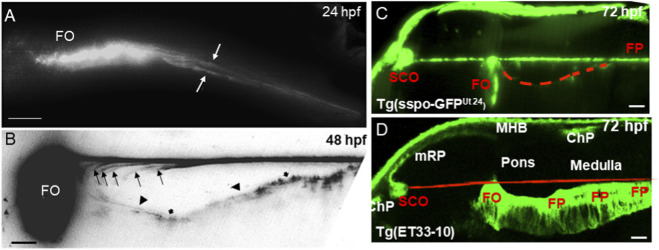
RF is connected to SCO and FO but separated from the hindbrain floor plate. **(A)** The AFRU-positive signal (shown in white color) is duplicated at the level of anterior hindbrain (24 hpf, white arrows). **(B)** The multiple fine layers of GFP-positive material (shown in black color; black arrows; the *scospondin-GFP* ^*ut24*^ transgenic line) were detected posterior to the FO at the level of anterior hindbrain (48 hpf; black arrowheads indicate the apical surface of the FP). The RF projects towards the central canal in a straight line. The hindbrain RF is disconnected from the apical surface of the FP. **(C)** Tg (Sspo-GFP), the white arrows show the weak GFP signal at the hindbrain FP apical surface (red dashed line); **(D)** ET33-10 (modified from García-Lecea et al., 2017; green - GFP; red - projected RF trajectory). In **(A,B)** The GFP signal is converted to black-and-white. Abbreviations: ChP–choroid plexus; E, epiphysis; FO, flexural organ; FP, floor plate; MHB, midbrain-hindbrain boundary; mRP, midbrain roof plate; SCO, subcommissural organ. N = 6 **(A–C)**. Scale bar, **(A,B)** = 20 μm; **(C,D)** = 10 μm.

Recent improvements in scientific research methods have prompted the reevaluation of previously studied biological processes (Muñoz et al., 2019). Combining whole-mount immunohistochemistry with light-sheet microscopy improves the analysis of macromolecular complexes during development, both *in vitro* and *in vivo*. We used this approach to reanalyze RF development in zebrafish. An early AFRU + signal was detected at 16 hpf in association with the anterior floor plate (not shown), which is known to form the FO. At 18 hpf, the intensity of this signal increased, as did the size of the area (Figure 1A). At 30 hpf, the floor plate-associated AFRU + signal in the hindbrain enlarged anteriorly and split into two lines posteriorly (Figures 1B–D; Figure 2A; Supplementary Figures S1B, C). This phenomenon was not reported previously.

*Scospondin-GFP* ^*ut24*^ transgenics enable imaging of RF formation *in vivo* (Troutwine et al., 2020). Two signals in the hindbrain were detected in *scospondin-GFP* ^*ut24*^ transgenic fish also (Figures 2B,C). The analysis revealed that the ventral signal is associated with the apical surface of the hindbrain floor plate, which bends ventrad (see Figures 2B–D). The dorsal signal represents the RF. During development, the anterior neural tube bends, while it continues to secrete pre-RF AFRU + material. The splitting of the signal is likely due to the ventral bending of the floor plate. During this process the deposited RF likely separates from the apical surface of the floor plate, which continues to secrete pre-RF AFRU + material that forms the additional signal.

During development, the anterior portion of AFRU + signal begins to stretch along the dorsal-ventral (D - V) axis (Figure 2A) (Placzek and Briscoe, 2005). As a result of the floor plate bending posterior to the midbrain-hindbrain boundary (MHB), the FO acquired the hook-like shape at 72 hpf (Figure 2D). High-resolution imaging of the hindbrain revealed the presence of the multiple GFP-positive threads between the two main signals (Figure 2B). These could be the result of AFRU + material deposition by the floor plate during continuous bending or individual RF microfilaments/groups of microfilaments that have separated during the floor plate bending.

**FIGURE 3:**
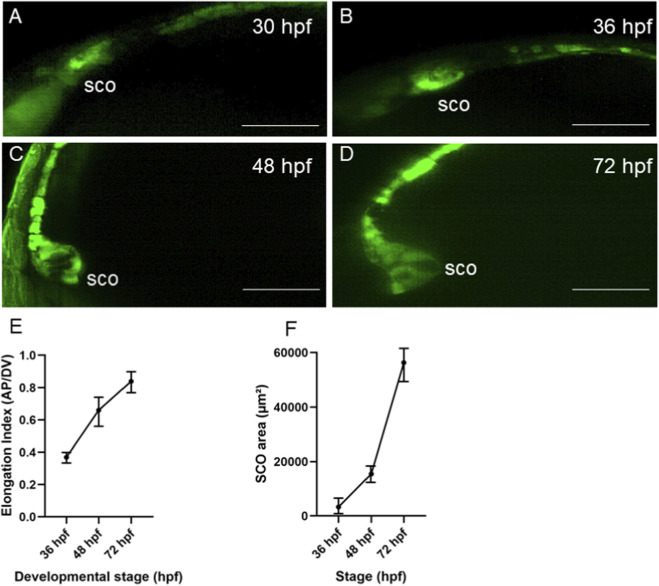
SCO morphogenesis in ET33-mi2a transgenics. **(A)** 30 hpf, **(B)** 36 hpf, **(C)** 48 hpf, **(D)** 72 hpf. All images are in the lateral view. **(E)** SCO elongation during development; **(F)** SCO area enlargement. For each embryo, the SCO area (µm^2^) was measured, and the antero-posterior (A-P) length and dorso-ventral (D-V) height were recorded to calculate the elongation index (A-P/D-V ratio). Values are presented as mean ± SD (n = 6 embryos per stage). MHB–midbrain-hindbrain boundary. Scale bar – 60 μm.

During the 30–36 hpf period, the diencephalic-mesencephalic roof plate forms the SCO. Initially, cells from the most anterior region of the roof plate extend along the D-V axis (Figures 1C,D; Figures 3A,B). This conclusion is supported by the analysis of the SCO elongation index (Figure 3E). As a result, the SCO reshapes into a triangular pocket (Figures 1C,D; Figures 3C,D) and its area increases significantly (Figure 3F). These measurements demonstrate that the SCO undergoes progressive enlargement and elongation between 36 and 72 hpf. Roof plate cells became AFRU + at 30 hpf (Figure 1B). By 36 hpf, the SCO-derived AFRU + thread connected the SCO and FO, illustrating the fusion of the anterior and posterior RF at the FO (Figure 1C). Anterior RF staining intensity increased during the 30–72 hpf period (Figures 1B–D). At 72 hpf, multiple SCO-derived branches fused at different anteroposterior (A-P) levels between the SCO and FO (Figure 1D).

**FIGURE 4:**
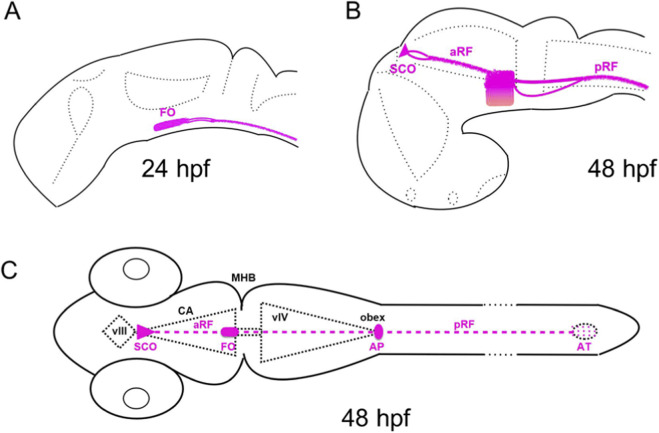
Schematic representation of the organs involved in formation of the RF. **(A)**, The FO/floor plate-derived RF (pRF) forms on the apical surface of the floor plate. The AFRU + signal is duplicated posterior to the FO with the dorsal signal representing the posterior RF and the ventral one representing the AFRU + material on the apical surface of the ventrally bending floor plate. **(B)** The SCO-derived RF forms in the cerebral aqueduct (CA). **(C)** Both parts of the RF join at the FO. Abbreviations: aRF, anterior Reissner fiber; AT, ampulla terminalis; FO, flexural organ; MHB, midbrain-hindbrain boundary; pRF, posterior Reissner fiber; SCO, subcommissural organ; vIII, third ventricle; vIV, fourth ventricle; wt, wild-type.

These results support an idea that RF forms step-by-step by the midline floor plate and SCO, respectively (Lehmann and Naumann, 2005; Meiniel et al., 2008). The hindbrain RF lost contact with the floor plate soon after being formed (Figures 4A,B). In contrast, the FO remained in contact with both the anterior and posterior RF (Figure 4B). Thus, the FO appears to function as a connecting hub for the two independently derived parts of the RF. The posterior RF, which is generated by the midline floor plate, is a transient structure that functions as a guide for the anterior RF, which is generated by the SCO. Thus, during RF development there is a transition from the ventral midline-derived posterior RF to the dorsal midline-derived anterior RF.

### The Kv2.1 channel regulates the development of RF

**FIGURE 5:**
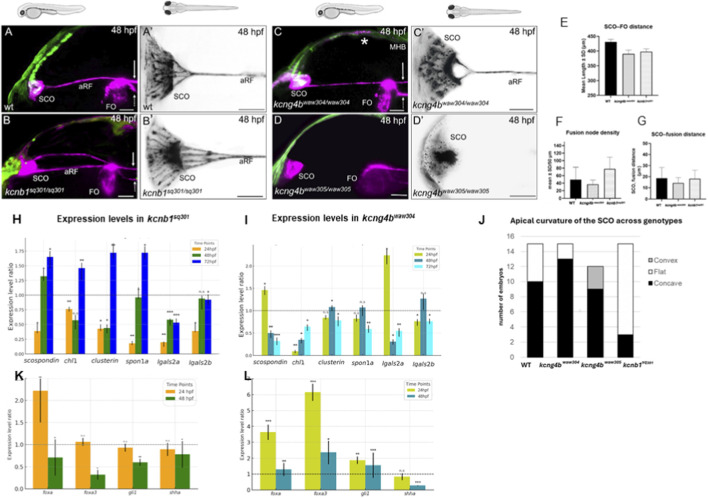
Mutations in the genes that encode the Kv channel subunits Kcnb1 and Kcng4b affect RF development. **(A)** In the wild-type 48 hpf embryo (n = 15), the anterior RF connects the SCO and FO. A′, Several SCO-derived filaments coalesce to form the anterior RF embryos (n = 15). The SCO apical surface was concave (10/15) or flat (5/15) (Table 1). **(B)** In the *kcnb1* ^*sq301/sq301*^ 48 hpf embryo (n = 15), the anterior RF was abnormally enlarged (9/15) or duplicated (6/15). B′, focus on the ventral anterior RF branch, where the number of SCO-derived filaments and fusion nodes increased compared to controls, and additional expression occurred in the roof plate anterior to the MHB. The SCO apical surface was concave (3/15) or flat (12/15). **(C)** The anterior RF was reduced in the *kcng4b* ^*waw304/waw304*^ 48 hpf embryo (n = 15). C′, The number of SCO-derived filaments was reduced to two with a single fusion node. The SCO apical surface was predominantly concave (13/15) or flat (2/15). **(D)** In the *kcng4b* ^*waw305/waw305*^ 48 hpf embryo (n = 15), the anterior RF was significantly reduced (3/15) or absent (12/15). D′, no SCO-derived filaments were detected. The SCO apical surface was concave (9/12) or convex (3/12) (Table 1). **(A–D)** Green - GFP expression in ET33-mi2a transgenics; magenta - anti-RF (AFRU) immunohistochemical staining detected by light sheet fluorescent microscopy (LSFM). A′-D′, color signals of GFP in the ET33-mi2a embryos and AFRU staining converted to the black-and-white; **(A–D)** lateral view, A′-D′, dorsal view. **(E)**, Length of anterior RF was measured in Fiji using the segmented line tool, from the subcommissural organ (SCO) apical margin to the anterior tip of FO. Values are mean ± SD. n = 6 embryos per genotype were analyzed, selected by QC for clear lateral views with full SCO–FO span. **(F)** Fusion node density (junctions per 50 µm). **(G)** SCO–fusion distance measured on the same QC subset (n = 6 per genotype). The node density weakly correlated with SCO–fusion distance (r = 0.22), indicating that filament branching complexity and fusion point positioning represent distinct parameters of anterior RF assembly. **(H,I)** Developmental profiling of expression levels of the RF-associated genes detected by quantitative RT-PCR in the *kcnb1* ^*sq301/sq301*^ **(G)** and *kcng4b* ^*waw304/waw304*^ **(H)** mutants. **(J)** The shape of the apical surface of the SCO in the controls and mutants. **(K,L)** The level of transcripts associated with ventral midline signaling in the *kcnb1* ^*sq301/sq301*^ **(K)** and *kcng4b* ^*waw304/waw304*^ **(L)** mutants. Expression values were normalized to wild-type embryos at each time point; this approach avoids misinterpretation caused by stage-dependent shifts in *sspo* expression domains. Scale bar - 20 μm. Abbreviations: aRF, anterior Reissner fiber; FO, flexural organ; MHB, midbrain-hindbrain boundary; SCO, subcommissural organ.

Mutations affecting the Kv2.1 subunits, Kcnb1 and Kcng4b, resulted in reduced or enlarged BVS, respectively (Shen et al., 2016). Since such changes affect RF development in mammals, the question arose as to whether these developmental defects affect RF formation in zebrafish and whether the circumventricular organs involved in RF formation, the SCO and FO, are affected. These organs originate from the signaling centers of the neural tube, i.e., the roof plate and midline floor plate. *kcnb1* ^*sq301*^ and *kcng4b* ^*waw304*^ and *kcng4b* ^*waw305*^ mutants were crossed with ET33-mi2a transgenics that express green fluorescent protein (GFP) in the SCO and FO. In the 48-hour-post-fertilization (hpf) wild-type controls, the SCO and FO were connected by a single line of anterior RF (Figure 5A) with the two parallel signals extending in the hindbrain posterior to the FO (Figure 5A, arrows). A dorsal view of the 48-hpf control embryo revealed a complex pattern of RF assembly from individual microfilaments. These microfilaments originated from the SCO apical surface (Figure 5A). The surface shape varied between concave (67%) and flat (33%; Table 1). The filaments fused at least twice before forming the single anterior RF. The staining also showed the complex radial distribution pattern of AFRU + material inside the SCO (Figure 5A’). To quantitatively define the microfilament fusion pattern, the SCO-FO distance (Figure 5E), fusion node density and distance between the SCO and the first fusion point (SCO-fusion) were calculated. Fusion node density was calculated as the number of junctions per 50 µm of filament length between the SCO and FO. In the controls this value was 49.0 ± 33.6 µm (Figure 5F), with an SCO-fusion distance of 18.6 ± 9.7 µm (Figures 5E,F).

In *kcnb1* ^*sq301*^ homozygotes at 48 hpf, the size of the midbrain cavity (*i.e.,* the cerebral aqueduct) was smaller than in the control group (Korzh, 2018; Shen et al., 2016). In this mutant, the two branches of the anterior RF appeared in a significant proportion of embryos with ectopic anterior RF in the dorsal position (lateral view, Figure 5B, arrowhead) or ventral position (Supplementary Figure S2A). This phenotype resembled that caused by Chl1a/Camel gain-of-function (Yang et al., 2021). The complex pattern of AFRU + material distribution in SCO was detected in these mutants. Posterior to the FO, the two parallel signals were present (Figure 5B, arrows). In a dorsal view, the four groups of two to four primary microfilaments originated from the largely flat apical surface of the SCO (n = 12/15; Figure 5J). The single thread of the anterior RF formed caudal to the SCO after at least three rounds of microfilament fusion. The SCO-FO distance was reduced compared with controls (Figure 5E). The fusion node density in these mutants (77.8 ± 31.5 µm) was significantly higher than in controls, and the SCO-fusion distance was similar to that in controls (18.0 ± 7.8 µm) (Figures 5F,G). *kcnb1* ^*sq301*^ mutants show increased fusion node density compared with wild type and changed curvature of the SCO’s apical surface (Figure 5J). These quantitative trends support the general observations (Figures 5A,B).

After observing the effects of *kcnb1* ^*sq301*^ on RF development, we investigated whether this mutation affects the transcript levels of genes associated with RF. Although the change in activity of this ion channel may have an indirect effect on transcription, this analysis suggests the potential avenues for the follow-up investigations and provides qualitative evidence to support the morphological data. At 24 hpf, *sspo* is only expressed in the midline floor plate (Meiniel et al., 2008). At this stage, *sspo* transcript level declined in the *kcnb1* ^*sq301*^ mutant compared to the wild-type control (Figure 5H). At 48 and 72 hpf, *sspo* expression in the floor plate declined during normal development, while increasing in the SCO (Meiniel et al., 2008). *sspo* expression levels in the *kcnb1* ^*sq301*^ mutant increased during this period compared to the wild-type controls (Figure 5H). This mutation appears to affect *sspo* transcript level differently in the floor plate and SCO. The levels of the *clh1a/camel*, *spon1a,* and *clu* transcripts have changed in the mutant, in a manner resembling the changes of *sspo* transcript level, with a significant increase at 72 hpf (Figure 5H). Galectin-1 acts as a partner of Scospondin during RF assembly in mammals (Muñoz et al., 2019). The expression pattern of galectin-1 (*lgals1*) has not been studied in developing zebrafish; however, two related genes (*lgals2a* and *lgals2b*) are expressed in the developing ependyma (Thijssen et al., 2006) and may be involved in BVS development. The *lgals2a* transcript level was significantly lower in *the kcnb1* ^*sq301*^ homozygotes than in the wild type controls at 24–72 hpf (Figure 5H).

In the *kcng4b* ^*waw304*^ mutant, which represents a hypomorphic Kcng4b loss-of-function allele (Jędrychowska et al., 2024), the BVS was enlarged compared to the wild-type control. The SCO-associated AFRU + filamentous network, as seen in the dorsal view, was less complex in this mutant compared to the wild-type control and *kcnb1* ^*sq301*^ homozygotes (Figure 5C, A’and B′). The anterior RF was present, but it formed after the fusion of two microfilaments derived from the predominantly concave apical surface of the SCO (n = 13/15; Table 1). The fusion node density in these mutants was lower than in controls (36.9 ± 11.1 µm) as was the SCO-fusion distance (14.2 ± 5.0 µm) (Figures 5F,G). The complex pattern of SCO staining was preserved in the *kcng4b* ^*waw304*^ mutant (Figure 5C). Quantitative analysis confirmed a measurable reduction in fusion node density and SCO–fusion distance in *kcng4b* ^*waw304*^ mutants compared with wild-type controls (Figures 5F,G). The phenotype of the *kcng4b* ^*waw305*^ allele (Jędrychowska et al., 2024) was more severe. The anterior RF was absent in some samples. This correlated with the significant reduction of the complex pattern of SCO staining and the convex apical surface of the SCO (Figures 5D D’ J). This phenotype resembles that caused by Chl1a/Camel loss of function (Yang et al., 2021). Yet the phenotype in majority of *kcng4b* ^*waw305*^ homozygotes was like *kcng4b* ^*waw304*^, i.e., with convex apical surface (Figure 5J). In contrast to the hypomorphic *kcng4b* ^*waw304*^, *kcng4b* ^*waw305*^ homozygotes exhibit reduced fertility and poor survival. Therefore, there was not enough material to perform the gene expression analysis on this line (see, Material and Methods).

The reduction of the filamentous network of anterior RF in the *kcng4b* ^*waw304*^ mutant correlated with the robust reduction in *sspo* transcript levels during the 48–72 hpf period, following a transient increase at 24 hpf (Figure 5I). While the *lgals2a* transcript level increased to 24 hpf, it decreased significantly by 48 and 72 hpf (Figure 5I). The level of the *clh1a/camel* transcripts significantly declined in contrast to that of *spon1a* and *clu* that were much less affected (Figure 5I). Thus, the transcript levels of *sspo* and *chl1a/camel* changed oppositely in the mutants affecting the two Kv2.1 subunits. At 24 hpf, the floor plate is the only source of *sspo*; however, by 72 hpf, *sspo* is predominantly expressed in the SCO (Meiniel et al., 2008). Therefore, the 24 hpf stage could be useful for evaluating the effect of Kv2.1 on the posterior RF using molecular markers expressed in the floor plate. The shortage of embryos prevented analysis of gene expression in homozygotes of the *kcng4b* ^*waw305*^ allele (see M&M).

The expression of genes acting downstream of Shh (*foxa3*, *gli1*) has been affected in the *kcnb1* mutant (Figure 5K). Foxa3 acting in concert with two other members of this family (Foxa1 and Foxa2) to positively regulate axial structures, including the floor plate (Dal-Pra et al., 2011). Notably, the *foxa3* transcript level increased significantly in the 24-hpf-old *kcng4b* ^*waw304*^ mutants (Figure 5L) unlike that in *kcnb1* ^*sq301*^ homozygotes (Figure 5K) suggesting a correlation between the activity of Kv2.1 and expression of floor plate markers. In contrast, the transcript level of the related gene *foxa*, which is mainly expressed in the neural crest and gut during this period (Odenthal and Nüsslein-Volhard, 1998), increased in both mutants (Figures 5K,L).

**FIGURE 6:**
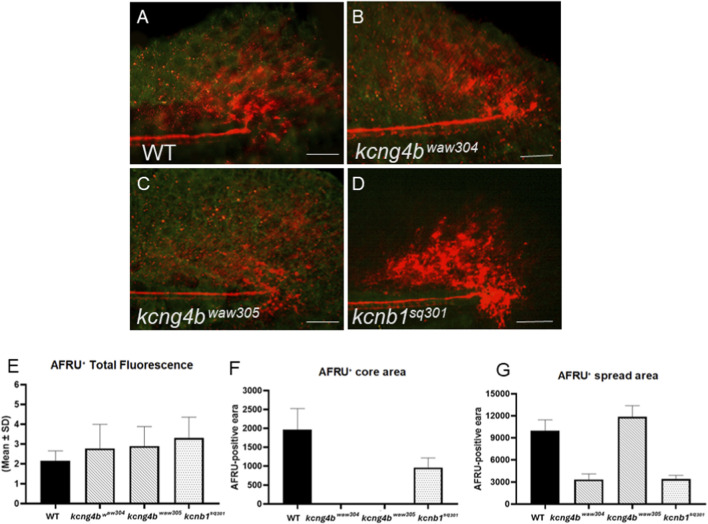
The mutations of Kv2.1 subunits affect RF disassembly in the ampulla terminalis (AT). The AFRU-stained AT in representative control **(A)**, *kcng4b* ^*waw304*^ **(B)**, *kcng4b* ^*waw305*^ **(C)**, and *kcnb1* ^*sq301*^ **(D)** embryos at 48 hpf. **(A–D)**–lateral view, anterior to the left-hand side. The AFRU + signal is red. The fluorescence of AFRU signal was quantified using ImageJ via thresholding. **(E)** The AFRU + total fluorescence; **(F)** The area of AFRU + intense fluorescence; **(G)** The total area of AFRU + spread (intense and diffuse regions). N = 6. Scale bar: 20 µm.

Defects in RF development are intrinsically linked to RF disassembly in the ampulla terminalis at the posterior end of the neural tube (Figure 4C) (Meiniel et al., 2008; Yang et al., 2021). The level of total fluorescence in this region increased in all mutants compared to wild type controls (Figure 6E). At the same time, the signal spread relatively broadly in the *kcng4b* ^*waw305*^ mutant (Figures 6C,H), in contrast to controls and *kcnb1* ^*sq301*^, where it seems to be more compact (Figures 6A,D,G). It seems that the Kcnb1-Kcng4b mutants affected not only the assembly of RF, but its disassembly also.

## Discussion

We conclude that mutations affecting the activity of Kv2.1 cause opposite changes in the expression of genes associated with RF formation, intracellular distribution of Sspo and its secretion, and the shape of the apical surface of the SCO. Previous studies have demonstrated the effect of changing the curvature of the cell membrane on Kv2.1 activity (Schmidt et al., 2012). A picture emerging from analysis of the SCO apical membrane in Kcnb1 and Kcng4b mutants suggested that changes in Kv2.1 activity modify the SCO apical surface topology (Figure 5). It is unclear whether this effect is direct. Whereas membrane tension regulates ion flow through the channel (Schmidt et al., 2012), the reverse process could be envisioned too. The membrane deformation could also be due to variations in the number and distribution of SCO-derived microfilaments that induce different tensions. In this case, initial deformation induced by microfilaments could trigger further changes in the activity of the mechanosensory mechanism (Porazinski et al., 2015; Takahashi et al., 2021; Voltes et al., 2019).

The effect of Kv2.1 seems to differ in the two sources of Sspo, i.e., midline floor plate and roof plate-derived SCO. It seems that Kv2.1 affects the formation of RFs at different levels, including expression of genes that encode RF components. Whether voltage-gated channels such as Kv2.1 are directly involved in regulation of transcription is unlikely. But as the principal component of ER-PM junctions involved in store-operated calcium entry, Kv2.1 may regulate activation of signal cascades, modification of transcription factors and transcription (Johnson et al., 2018; Zarain-Herzberg et al., 2011).

The changes in Kv2.1 activity have been associated with developmental defects of the BVS (Shen et al., 2016), which as shown by studies of the RF development in *hyh* mice may impact RF formation (Pérez-Fígares et al., 1998; Wagner et al., 2003). The results presented demonstrate that the defects of the BVS’ spatial expansion affect RF development in zebrafish in a manner suggesting the Kv2.1 negative control over Sspo. This control may take place at different levels, including *sspo* expression and secretion, leading towards SCO and RF formation, and RF disassembly. Given that Kv2.1 is involved in the intracellular protein traffic (Jędrychowska and Korzh, 2019; Jensen et al., 2017) and insulin secretion (Jacobson et al., 2007), it is possible that Kv2.1 regulates Sspo secretion by cells producing RF proteins. The question is whether Kv2.1 effect is the same in these two different lineages, *i.e.*, the midline floor plate and roof-plate-derived SCO cells representing the two main signaling centers of the neural tube.

The difference in the developmental expression profiles of *sspo* in the floor plate and SCO allows to discriminate to some extent the tissue-specific expression of this gene using the whole-body qRT-PCR. At 24 hpf only the floor plate expresses *sspo*, whereas later the floor plate expression is downregulated. In result, at 72 hpf the SCO is responsible for the bulk of *sspo* expression (Meiniel et al., 2008). Reduced Kv2.1 activity in *kcnb1* mutants (Jędrychowska et al., 2024) correlates with the decreased level of *sspo* transcript at 24 hpf, i.e., the time point *sspo* expression is confined to the floor plate (Figure 5H). In line with this result, increased Kv2.1 activity in *kcng4b* mutants correlates with an increase of the *sspo* transcript level (Figure 5I). In contrast, at 72 hpf, the opposite situation was detected (Figures 5H,I). Therefore, Kv2.1 likely may have an indirect positive effect on the *sspo* transcript level in the floor plate and an opposite effect on that in the SCO. This assumption is supported by the increase of the AFRU + signal associated with the anterior RF in *kcnb1* mutants and its reduction and/or absence in *kcng4b* mutants (Figures 5B–D). Thus, Kv2.1 subunits exert antagonistic effects on BVS enlargement, as previously demonstrated (Shen et al., 2016). Within this process, Kv2.1 subunits have different effects on BVS signaling centers, such as the roof and floor plates.

Mutations in Kv2.1 subunits also affect the levels of several other transcripts implicated in RF development. Interestingly, changes of Kv2.1 activity correlate with expression level and function of another regulator of RF formation - *chl1a/camel* (Figures 5H,I). The gain of function of Kv2.1 and Chl1a/Camel causes the formation of ectopic RF, though in Kv2.1 mutants the defects seem localized to the third ventricle and cerebral aqueduct, unlike defects caused by manipulation of Chl1a/Camel levels, which spread into the hindbrain. Kv2.1 and Chl1a/Camel loss-of-function results in RF reduction or absence (Figures 5B–D) (Yang et al., 2021). Taken together, these results reveal the complex nature of Kv2.1’s effects on RF formation. It could be intrinsically linked to the development of BVS as a whole (Pérez-Fígares et al., 1998; Shen et al., 2016; Wagner et al., 2003). However, the effect of Kv2.1 on specific elements of BVS differs and likely extends beyond membrane ion flow. This may result from other Kv2.1 functions, such as intracellular protein transport, the formation of ER-plasma membrane junctions, the maintenance of the plasma membrane potential, and the formation of cholesterol-enriched lipid rafts (Deutsch et al., 2012; Johnson et al., 2019; Tamkun et al., 2007).

The RF forms in several steps. First, the midline floor plate, which is derived from the embryonic shield, transiently secretes Scospondin, forming the posterior RF along nearly the entire neural tube from the midbrain to the neural tube’s most posterior tip (Halpern et al., 1997; Meiniel et al., 2008; Shih and Fraser, 1996; Strähle et al., 2004). Thus, the posterior RF, which is transient and derived from the midline floor plate, is an acellular structure generated by the cells originating from the embryonic organizer. As a transient embryonic structure, the posterior RF acts as a template organizing the SCO-derived anterior RF and extending in such a way the role of the embryonic organizer. Furthermore, the FO is not only an attachment point for the RF. It also connects the anterior and posterior parts of the RF. Thus, the early input from the embryonic organizer can be traced all the way to the RF, which coordinates information on body movements along the body axis (Driever, 2018).

Floor plate cells located anterior to the MHB give rise to FO (Figures 1A–C). Unlike the rest of the posterior floor plate, this circumventricular organ maintains contact with the RF. The second circumventricular organ, the SCO, then forms the anterior RF (Figures 1A–C). The anterior RF connects to the FO and, over time, replaces the posterior RF derived from the floor plate. Therefore, the posterior RF, derived from the embryonic shield, acts as a template for the definitive “anterior” RF.

The molecular mechanism responsible for the induction of *sspo* expression in roof plate-derived SCO likely relies on the ventralization of the diencephalon. The process seems to depend on rapprochement of the ventral and dorsal signaling centers (roof plate and floor plate) at the mid-diencephalic boundary, which results in the expression of downstream Shh pathway components, such as Smo in the SCO and Isl1 in the pineal gland and nuclei of the posterior commissure (Baeuml et al., 2019; Korzh et al., 2007; Korzh et al., 1993; Traiffort et al., 1999).

Although the effect of Kv2.1 mutations on gene expression is likely indirect, its quantitative evaluation provided valuable insights, particularly in the context of morphological changes caused by mutations. RF development relies on the coordinated activity of many genes that encode proteins that form the RF *(sspo, lgals1, clu, chl1a/camel, etc*.) (Cantaut-Belarif et al., 2018; Muñoz et al., 2019; Yang et al., 2021). RF development also depends on BVS developmental regulators such as the α-SNAP deficient in *hyh* mutant mice (Pérez-Fígares et al., 1998; Wagner et al., 2003). Interestingly, the BVS phenotype of zebrafish *kcng4b* mutants resembles that of *hyh* mice (Shen et al., 2016). Developmental changes in the BVS of zebrafish mutants deficient in the Kv2.1 subunits suggest that Kv2.1 activity negatively regulates RF formation and disassembly (see Figures 5, 6). This appears to be due to changes in the transcript levels of genes involved in RF formation, such as *sspo*, *lgals2a* and *chl1a/camel* (Figures 5H,I). The formation of RFs emerges as a chain of complex events, and Kv2.1 appears to be one of the factors that regulate this process.

This analysis raised some questions that could be answered by follow-up studies. The formation mechanism of the duplicate anterior RF in Kcnb1-deficient embryos remains unknown. This phenomenon could be linked to the premature closure of the cerebral aqueduct, which is caused by a reduction of the BVS, and the formation of the two separate channels for CSF circulation (Fame et al., 2016). However, there is a lack of clear evidence. Given the multitude of roles of Kcnb1 discussed above, it would be interesting to determine which role—maintaining the membrane potential, trafficking secreted proteins, or regulating secretion at the plasma membrane or all of these—could be involved in the development of the anterior RF.

## Data Availability

Publicly available datasets were analyzed in this study. This data can be found here: GEO submission no. GSE194272.

## Supplementary material

SUPPLEMENTARY FIGURE S1 The cross section of the 48 hpf wildtype embryo at mid-hindbrain (ear) level. A – bright field image; B, C – confocal images of the same specimen. Green, AFRU antibody, red, anti-F-actin (phalloidin) staining. FP – floor plate; N – notochord, NT – neural tube, OV – otic vesicle, Y – yolk. Scale bar – 20 µm.

SUPPLEMENTARY FIGURE S2 Some Kcnb1 mutants develop duplicated or enlarged anterior RF. A, duplicated anterior RF; B, enlarged anterior RF (lateral views). SCO, subcommissural organ, FO, flexural organ, RF, Reissner fiber. Green, GFP expression in the choroid plexus of the compound *kcnb1* ^*sq301*^/Tg (ET33-20) embryos. Scale bar – 20 µm.
