# Supplementary figures and images for: Kv2.1 negatively regulates Reissner fiber development
Source: Front Cell Dev Biol. 2026 Jan 5;13:1720752. doi: 10.3389/fcell.2025.1720752 (PMC12812919; doi:10.3389/fcell.2025.1720752)

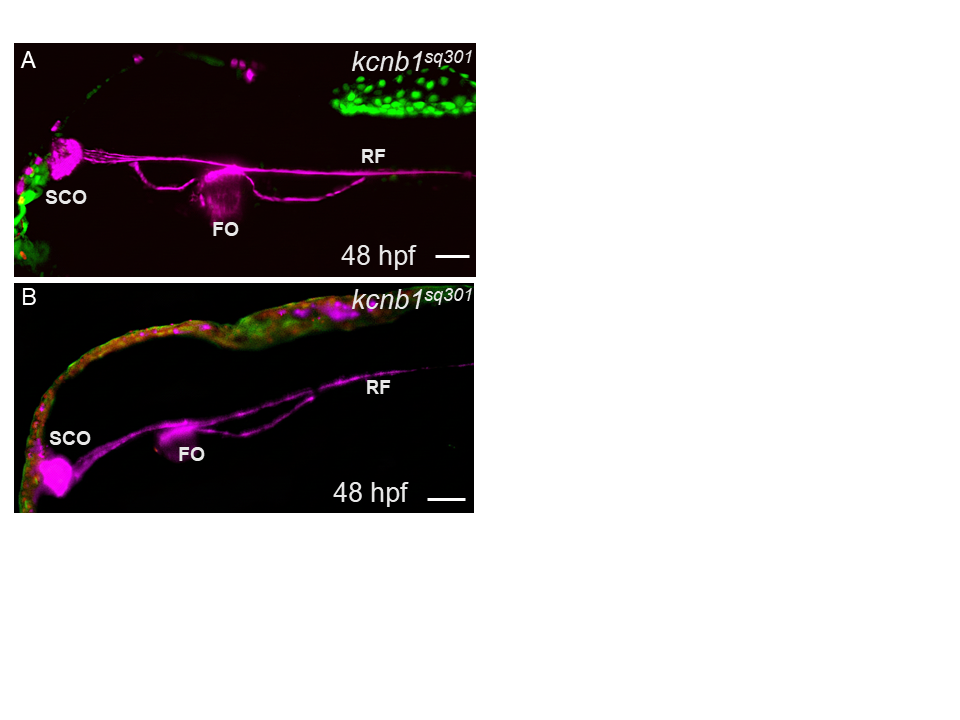

Supplement: Supplementary file 1 [file Image2.tif]

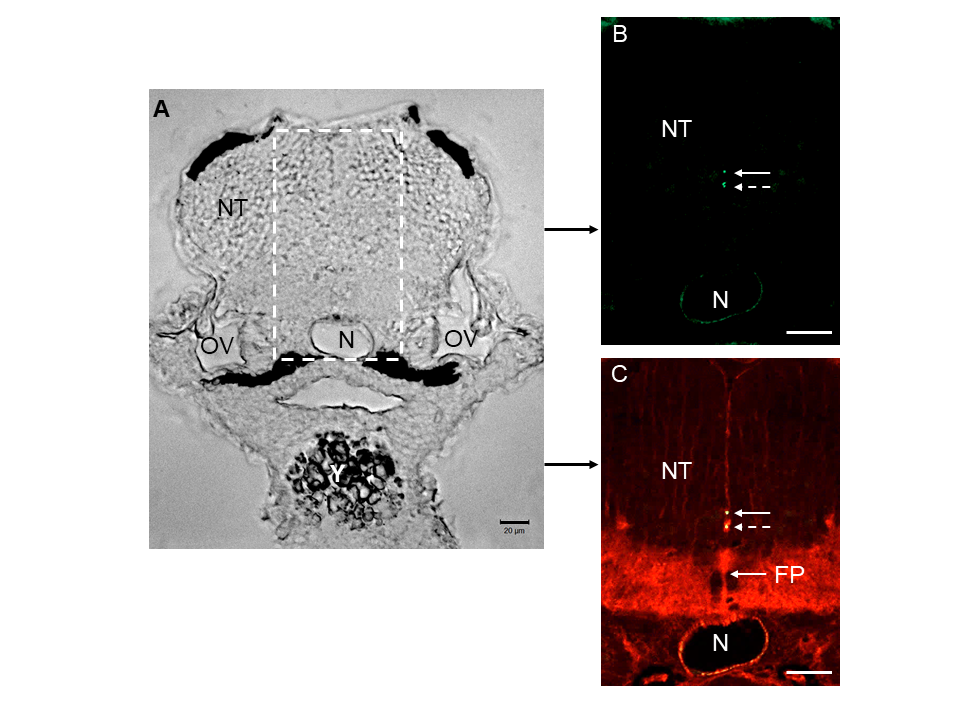

Supplement: Supplementary file 2 [file Image1.tif]
